# 

**Published:** 1893-01-28

**Authors:** 


Per Annum, 8s. 6d., post free.

Monthly Parts, 6d.; post free, 8d.

Ditto per Annum; 8s., post free.

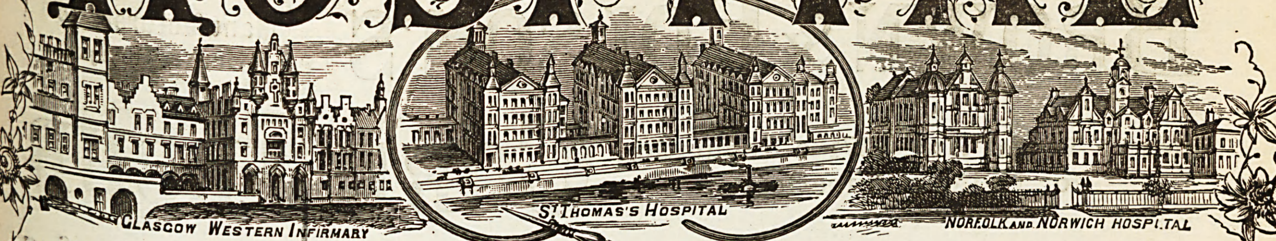

WITH EXTRA NURSING SUPPLEMENT.

No. 331. Vol. XIII.] SATURDAY, JANUARY 28th, 1893. [TWOPENCE.

